# Dovetailing biology and chemistry: integrating the Gene Ontology with the ChEBI chemical ontology

**DOI:** 10.1186/1471-2164-14-513

**Published:** 2013-07-29

**Authors:** David P Hill, Nico Adams, Mike Bada, Colin Batchelor, Tanya Z Berardini, Heiko Dietze, Harold J Drabkin, Marcus Ennis, Rebecca E Foulger, Midori A Harris, Janna Hastings, Namrata S Kale, Paula de Matos, Christopher J Mungall, Gareth Owen, Paola Roncaglia, Christoph Steinbeck, Steve Turner, Jane Lomax

**Affiliations:** 1 Mouse Genome Informatics, The Jackson Laboratory, Bar Harbor, ME 04609, USA; 2 The Gene Ontology Consortium, Bar Harbor, ME, USA; 3 Department of Genetics, University of Cambridge, Downing Street, Cambridge, CB2 3EH, UK; 4 University of Colorado Anschutz Medical Campus, Aurora, CO, USA; 5 Royal Society of Chemistry, Thomas Graham House, CB4 0WF, Cambridge, UK; 6 The Arabidopsis Information Resource, Carnegie Institution for Science, Stanford, CA 94305, USA; 7 Current address: Cambridge Systems Biology Centre and Department of Biochemistry, University of Cambridge, Sanger Building, 80 Tennis Court Road, CB2 1GA, Cambridge, UK; 8 European Molecular Biology Laboratory - European Bioinformatics Institute, Wellcome Trust Genome Campus, Hinxton, CB10 1SD, Cambridge, UK; 9 Genomics Division, Lawrence Berkeley National Laboratory, Berkeley, CA, USA; 10 current address: CSIRO Materials Science and Engineering, Bayview Avenue, Victoria 3168, Clayton, Australia

## Abstract

**Background:**

The Gene Ontology (GO) facilitates the description of the action of gene products in a biological context. Many GO terms refer to chemical entities that participate in biological processes. To facilitate accurate and consistent systems-wide biological representation, it is necessary to integrate the chemical view of these entities with the biological view of GO functions and processes. We describe a collaborative effort between the GO and the Chemical Entities of Biological Interest (ChEBI) ontology developers to ensure that the representation of chemicals in the GO is both internally consistent and in alignment with the chemical expertise captured in ChEBI.

**Results:**

We have examined and integrated the ChEBI structural hierarchy into the GO resource through computationally-assisted manual curation of both GO and ChEBI. Our work has resulted in the creation of computable definitions of GO terms that contain fully defined semantic relationships to corresponding chemical terms in ChEBI.

**Conclusions:**

The set of logical definitions using both the GO and ChEBI has already been used to automate aspects of GO development and has the potential to allow the integration of data across the domains of biology and chemistry. These logical definitions are available as an extended version of the ontology from http://purl.obolibrary.org/obo/go/extensions/go-plus.owl.

## Background

New high-throughput technologies are being used to generate large quantities of data detailing interactions between genes, molecules and biological systems. There is a critical need for informatics tools that can integrate both chemical and biological knowledge with these datasets to gain a deeper understanding of biological networks [1] and to stimulate drug discovery [2]. One informatics resource that has transformed the analysis of large biological datasets is the Gene Ontology (GO) [3], which provides a computable description of the functional aspects of an increasing number of genes and gene products spanning a diverse range of species. The GO is used by gene product annotators to assign attributes to protein and functional RNA gene products based on experimental reports in the primary literature [4-12]. These annotation sets are used for large dataset interrogation to determine similarities and differences in the attributes of gene products within those datasets. For example, the GO has been used to look into the function of genes that may contribute to adaptation of humans to high altitudes as well as to find correlations between gene function and diseases or disorders such as cancer or autism [13-16].

At the core of the GO is a collection of over 36,000 terms, connected by more than 67,000 relationships. For ontology curators to maintain accuracy and consistency in a structure this large and complicated, it is critical to incorporate automated methods to check for integrity and to help build the ontology graph [17-19]. To this end, the GO has been adding computable definitions for many of its terms [19]. Automatic reasoners can then use these logical definitions to check for logical inconsistencies in the graph, to infer additional relationships, and to automatically classify new terms within the hierarchy. This approach becomes even more powerful when the definitions include components both from within the GO and from specialized external ontologies, as it allows for the resources to build on the information contained within the other ontologies.

A complementary resource for chemists is ChEBI (Chemical Entities of Biological Interest). ChEBI consists of approximately 30,000 terms organized along similar principles to the GO [20]. ChEBI acts as a reference for chemical entities across multiple domains and includes in its repertoire a wide range of entities from the subatomic scale to complex polymers. A growing number of bioinformatics resources are utilizing both the GO and ChEBI as part of a substantial effort to integrate a large set of biological ontologies for community use [21-26].

The GO and ChEBI have been constructed largely for different purposes and, until recently, have only been loosely connected. Previous efforts to align information between these resources have used semantic similarity and string matching methods [27,28]. Here we describe the first stages of a process to fully dovetail the two ontologies, achieved through a combination of automated tools and the considerable efforts of domain experts in biology, biochemistry and chemistry working to reconcile multiple organizational views to achieve an integrated whole. As a result of this work, we have new interoperable versions of each of these two ontologies and a collection of relationships that bridge them. This combined resource has already been used to automatically generate and classify new terms within the GO using a Web Ontology Language (OWL) representation of the GO in conjunction with some specifically designed tools like the OBO Ontology Release Tool (Oort, http://code.google.com/p/owltools/wiki/OortIntro) and TermGenie (http://go.termgenie.org/, Dietze et al., manuscript in preparation), which make use of powerful OWL reasoners such as ELK and HermiT [29,30]. We hope that this approach and the lessons learned during our collaboration will provide other ontology developers with a basic roadmap to integrating other ontologies more efficiently.

## Results and discussion

### Inherent GO chemical ontology

**Figure 1:**
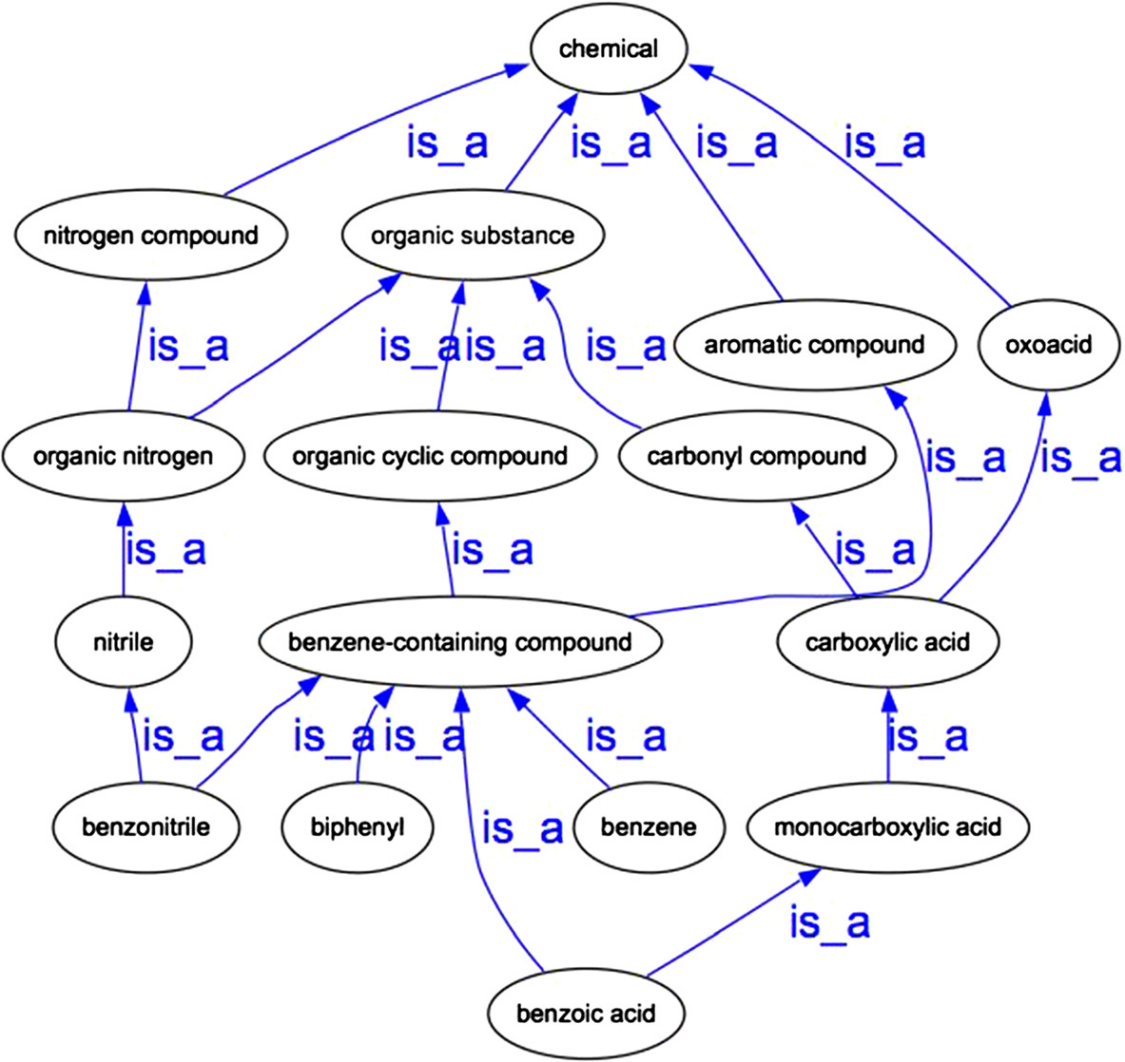
**An example of a portion of the GO chemical ontology representing the inherent chemical ontology within the GO.** The entire ontology consists of the union of all possible inter-chemical relationships inferred by GO terms. This ontology was used as an initial step for curators to examine how chemicals were represented throughout the ontology.

Before comparing the GO to an external chemical ontology, the inherent chemical representations in the GO were checked for internal consistency. We identified inconsistencies that existed within the GO using a combination of automated and manual methods. First, we generated a graph in which the chemicals in the GO were separated from processes and functions and represented as stand-alone chemicals with relationships that followed the *is*_*a* hierarchy in the GO (Figure 1). This allowed us to extract the implicit chemical ontology embedded within the GO.

We determined that there were eight sub-ontologies in the GO where chemical names were frequently used: ‘response to chemical stimulus’ (GO:0042221), ‘transport’ (GO:0006810), ‘transporter activity’ (GO:0005215), ‘metabolic process’ (GO:0008152), ‘biosynthetic process’ (GO:0009058), ‘catabolic process’ (GO:0009056), ‘catalytic activity’ (GO:0003824) and ‘binding’ (GO:0005488), as well as the sub-ontologies representing types of regulation of these processes and functions. When possible, these chemical names were cross-referenced to the chemicals in ChEBI. Relationships in this ‘GO chemical ontology’ represented the union of all of the *is*_*a* relationships for each sub-ontology of the GO.

**Figure 2:**
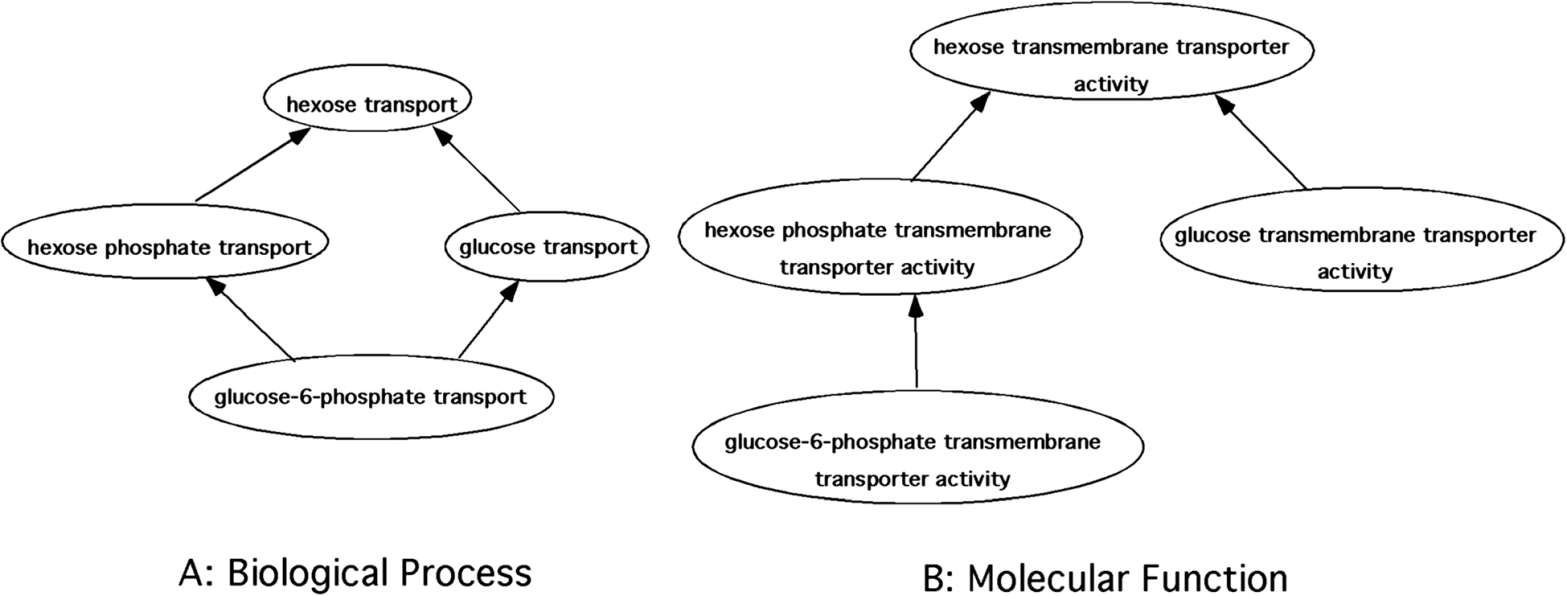
**Example of inconsistencies of chemical representations within the GO.** Arrows represent is_a relationships between terms. **A)** The representation of **‘glucose-6-phosphate’** in the transport portion of the biological process graph. **‘Glucose-6-phosphate’** is inherently both a **‘glucose’** AND a **‘hexose phosphate’ B)** The representation of **‘glucose-6-phosphate’** in the transporter portion of the molecular function graph. **‘Glucose-6-phosphate’** is inherently a **‘hexose phosphate’** but NOT a **‘glucose’**. Only relevant terms and their relationships are shown.

Previous work in deductive and abductive reasoning over relationships asserted between terms from the GO and other ontologies has revealed a large number of inconsistencies within the GO itself. For example, inconsistent representations of chemicals were found among biological processes and molecular functions (Figure 2) [31]. This type of error and inconsistency identified in the GO’s representation of chemicals during manual review were corrected. This corrected GO chemical ontology was then used as a template for coordination with ChEBI.

### Differences between the GO and ChEBI

Adjustments resulting in concordance of the implicit GO chemical ontology with the ChEBI ontology required two processes. Initially, cross-references between the GO and ChEBI were generated by a string-matching algorithm between GO terms and synonyms and ChEBI terms and synonyms. Those cross-references were then checked manually during the curation of the GO chemical ontology. Terms that were not cross-referenced—that is, the implicitly represented chemicals in the GO terms for which corresponding terms in ChEBI were not found—fell into three categories:

1. Terms in the GO chemical ontology that referred to existing terms in the ChEBI ontology, but for which the matches were not detected automatically due to naming differences. During this analysis both term names and synonyms are utilized to determine matches. For example, the GO chemical ‘**butanoic acid**’ should be cross-referenced to the ChEBI entry ‘**butyric acid**’ (CHEBI:30772). These terms were identified and cross-referenced manually.

2. Chemicals in GO terms that were not represented in ChEBI. 283 temporary terms with internal identifiers were created to represent these chemicals, which have subsequently been incorporated into ChEBI, where appropriate, via the ChEBI online submission tool [20].

3. Terms that represented axes other than that of structural chemical classification, e.g., function, disposition or role axes of classification.

### Resolution of differences between the GO and ChEBI

In addition to cases where chemicals implicitly represented in GO terms were not represented in ChEBI, we also found cases where the chemicals were represented in both the GO and ChEBI, but the representation of chemicals did not match due to differences in the terms themselves or in the hierarchical relationships. These discrepancies reflect deeper differences in the use of chemical terminology and classification between biologists and chemists.

### Case 1: Singular vs. plural terminology

The use of plurals in class names is not generally recommended in ontology construction because it can introduce ambiguity in the meaning of a term and cause problems with string matching when used in conjunction with other ontologies [32]. However, ChEBI uses plural term names to designate families of structurally related molecules that are named after a specific molecule from which they are formally derived. Plural names used in this way follow International Union of Pure and Applied Chemistry (IUPAC) recommendations and are therefore the accepted way for chemists to refer to these families of structures. For example, ‘**pyridines**’ (CHEBI:26421) encompasses all molecules that contain a pyridine ring, whereas ‘**pyridine**’ (CHEBI:16227) specifically represents the molecule that contains a pyridine ring and has the formula C5H5N. In vernacular English usage, however, a singular noun such as ‘pyridine’ can be ambiguous between the specific sense, corresponding to either ‘**pyridine**’ in ChEBI (CHEBI:16227) and to the EXACT reading in the notation of Corbett *et al*[33], or the collective sense corresponding to ‘**pyridines**’ in ChEBI (CHEBI:26421) and the CLASS reading. As a result of this ambiguity, the GO contained definitions that refer to both CLASS readings, as in ‘**phenol metabolic process**’ (GO:0018958), and to EXACT readings, e.g. ‘**benzene metabolic process**’ (GO:0018910).

We decided to reconcile this difference in nomenclature by renaming the plural terms as '**X**-**containing compound**' for GO chemicals. In the example above, GO:0018958 is renamed '**phenol**-**containing compound metabolic process**'. Terms of equivalent meaning to the plural terms in ChEBI can now easily be created in the GO. So far, 86 terms in the GO have been renamed to reflect the new convention.

### Case 2: Representation of complex molecules such as nucleotides and nucleosides

The representation of complex molecules such as nucleotides, nucleosides or liposaccharides was another area that posed problems for the coordination of ChEBI and the GO. The issue arose because there are two main strategies used by chemists to classify molecules. The natural product strategy predates the acceptance of the atomic theory by organic chemists and involves classifying molecules according to their biological histories; hence alkaloids were those nitrogenous molecules extracted from plants, terpenoids are those molecules that have been derived from terpene molecules by chemical modification and so forth. They share a family resemblance, although there is no single structural signature for a given class that allows for its classification. The functional strategy, conversely, involves identifying functional groups, such as carboxy groups or carbon–carbon double bonds within a molecule and assigning the molecule to a functional class such as, in this case, the carboxylic acids or the olefins, respectively. The choice of the name 'functional' is significant because the functional groups pick out those parts of a molecule that are disposed to react in a particular way. But because there is no limit to the number of functional groups within a molecule, there is in principle no limit to the number of functional classes to which a molecule can belong. From a structural point of view, describing these complex chemicals is similar to the story of the blind men and the elephant, where each man describes the elephant as something different depending upon the part he is touching. That is, classification is based on specific parts of the molecule that are attached at different positions. For example, a nucleotide that contains a nucleobase, a sugar and at least one phosphate group would be described as a carbohydrate by a carbohydrate biochemist, who is primarily concerned with the reactivity of the carbohydrate moiety of the molecule, whereas general organic chemists might classify it as a phosphoric ester.

Both of the above classifications are correct chemically, but they can lead to incorrect inferences when extrapolated to the process hierarchy in the GO. Consider ‘nucleotide metabolic process’ (GO:0009117), ‘carbohydrate metabolic process’ (GO:0005975) and ‘phosphate metabolic process’ (GO:0006796) in the GO. If ‘nucleotide metabolic process’ (GO:0009117) were classified as both *is*_*a* ‘carbohydrate metabolic process’ (GO:0005975) and *is*_*a* ‘phosphate metabolic process’ (GO:0006796) to parallel the structural hierarchy in ChEBI, then the process that results in the addition of a phosphate group to a nucleotide diphosphate would be misleadingly classified as *is*_*a* ‘carbohydrate metabolic process’ (GO:0005975). This is misleading because, since the carbohydrate portion of the nucleotide is not being metabolized, biologists would not typically consider this to be a carbohydrate metabolic process.

To avoid this kind of misleading inference, the constituent parts of a nucleotide in ChEBI are now represented using has_functional_parent assertions instead of explicitly asserted is_a relationships. ChEBI uses the has_functional_parent relationship to denote the relationship between two molecular entities (or classes of entities), one of which possesses one or more characteristic groups and from which the other can be derived by functional modification. Thus a nucleoside will be defined as ‘an *N*-glycosyl compound that has both a nucleobase and either a ribose or deoxyribose as functional parents’. As an extension, both ChEBI and the GO will define a nucleotide as ‘a phosphate ester that has a nucleoside moiety AND has at least one phosphate moiety attached to the C-5 carbon of the ribose or deoxyribose moiety’.

### Case 3: Representation of acids and their conjugate bases in the GO and ChEBI

**Figure 3:**
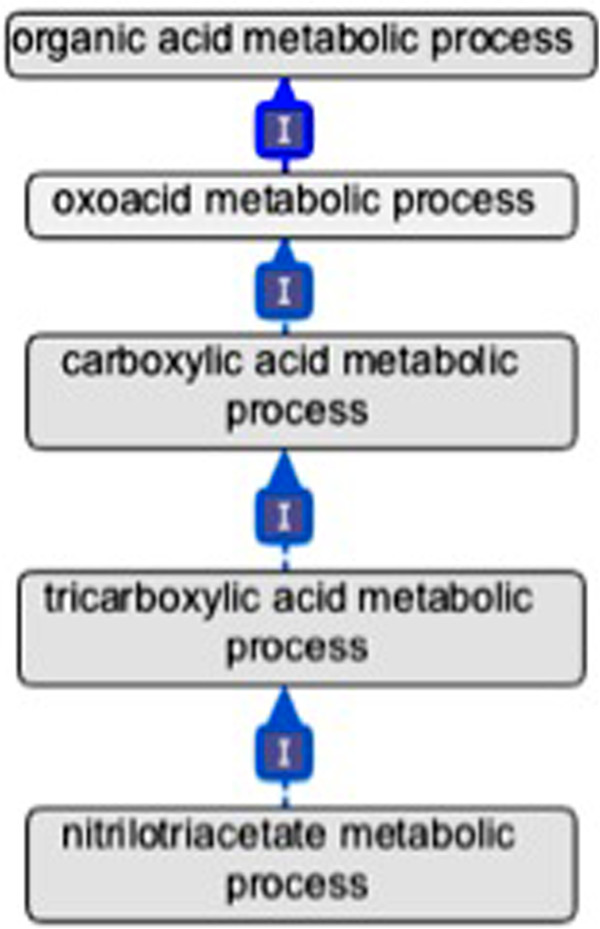
**An illustration of the combined usage of acid/conjugate base terminology in the GO.** In the GO, **‘nitrilotriacetate metabolism’** is a type of **‘tricarboxylic acid metabolism’.** This inherently states that nitrilotriacetate is a type of tricarboxylic acid. The underlying structure of the ontology now represents **‘nitrilotriacetate metabolism’** as **‘nitrilotriacetic acid’** metabolism and the metabolism of its conjugate bases. Only relevant terms and their relationships are shown.

Another area where the GO chemical ontology and ChEBI failed to agree was in the representation of acids and their conjugate bases. ChEBI has a detailed representation of acids and conjugate bases with the inclusion of ‘is_con-jugate_acid_of’ and ‘is_conjugate_base_of’ relationships in the ontology. So ‘carboxylic acid’ is_conjugate_acid_of ‘carboxylic acid anion’ and ‘carboxylic acid anion’ is_conjugate_base_of ‘carboxylic acid’. In contrast, in the GO, acids and their conjugate bases are often conflated. For example, in the GO ‘nitrilotriacetate metabolic process’ *is*_*a* ‘carboxylic acid metabolic process’ (Figure 3). The implicit GO chemical ontology therefore states that ‘nitrilotriacetate’ *is*_*a* ‘carboxylic acid’. From a chemical viewpoint, this is incorrect. ‘Nitrilotriacetate’ is the conjugate base of a member of the class ‘carboxylic acid’, ‘nitrilotriacetic acid’. This inaccurate representation in the GO reflects the conflated use of nomenclature for acids and their conjugate bases in the biological community because these molecules often readily interconvert as they participate in biological processes. To resolve this issue we have decided to combine the terminology for oxoacids and their conjugate bases in the GO, and make these combined entities equivalent to the union of the acid with its conjugate base or bases in an internal file, BioChEBI (see Methods below). This reflects the convention that when ‘acids’ are referred to in the biochemical literature, they most often refer to molecules that are playing the role of an acid and as such are deprotonated at physiological pH. It also allows for the integration of the GO chemical ontology, which is blind to the conjugate acid/base distinction, with a single *is*_*a* hierarchy in ChEBI.

A similar strategy could also be used for terms in other areas of ChEBI where two chemicals are related to each other using a non-*is*_*a* relationship. For example tautomers in ChEBI are distinguished by the relationship *is*_*tautomer*_*of* and could be treated in a similar manner as the acids and bases. However, we did not find it necessary to implement this strategy since we did not find a confusion of tautomeric structures in the inherent GO chemical structure. Similarly stereoisomers and salt forms did not present a problem. Stereoisomers were already distinguished in the GO and the corresponding ChEBI terms were mapped 1:1.

### Logical definitions for GO terms using ChEBI terms

GO terms were deconstructed and represented as intersections between GO terms and relationships to ChEBI terms. For example, ‘**carbohydrate binding**’ (GO:0030246) is represented as the intersection of the generic GO molecular function class ‘**binding**’ (GO:0005488) and a *has*_*input* relationship to the ChEBI class ‘**carbohydrate**’ (CHEBI:16646), while ‘**carbohydrate metabolic process**’ (GO:0005975) is represented as the intersection of the generic GO biological process class ‘**metabolic process**’ (GO:0008152) and a *has*_*input* relationship to the ChEBI class ‘**carbohydrate**’ (CHEBI:16646). As of mid-October 2012 there are 4,403 terms in the GO that have been defined using ChEBI chemical entities (Table 1).

**Table 1:** Terms in the GO that refer to chemicals

**Term type**	**Number of terms with logical definitions that refer to ChEBI**
chemical secretion	38
chemical binding	345
chemical transport	466
chemical metabolic process	1,120
chemical biosynthetic process	990
chemical catabolic process	870
response to chemical	196
chemical homeostasis	33
chemical transporter activity	324

The logical definitions of GO terms that reference chemical entities in ChEBI are maintained in an extended version of the ontology (http://purl.obolibrary.org/obo/go/extensions/go-plus.owl). When a new GO term is requested that involves a chemical entity not currently described in ChEBI, that chemical entity is first given to ChEBI to be reviewed for inclusion. Only when the new class is subsequently added to ChEBI is the corresponding GO term created. It will not be necessary to incorporate every ChEBI term into the GO hierarchy because many ChEBI terms will not be relevant in the description of natural biological processes. For example, the ChEBI term ‘**nucleoside triphosphate analog**’ (CHEBI:37413) which includes many chemicals used in biochemical inquiry, but will likely not be incorporated into the GO because they do not play roles in natural biological processes.

## Conclusion

In this manuscript we have described the process of resolving two independently developed ontologies for the purpose of knowledge integration and sharing. The integration of ChEBI and the GO benefits both ontologies since it allows for the consistent and accurate representation of chemicals in the GO, and for the chemicals represented in ChEBI to be placed in the natural biological contexts of GO processes. This interoperability of ontologies is one of the primary goals of the coordinated development of the group of ontologies that make up the OBO Foundry [21].

As a result of this work and the close ties that were established between the GO and ChEBI during this process, all new GO terms that involve the transport, metabolism, response to chemical entities and homeostasis can be added to the ontology via a new web-based tool called TermGenie (http://go.termgenie.org/, Dietze et al., manuscript in preparation). TermGenie is a template-based, reasoner-assisted term-generation tool. Annotators can generate these terms directly by selecting the broad GO category (e.g. transport, biosynthesis) and any term from ChEBI. Missing ChEBI entities need to be requested from ChEBI before proceeding. Labels, synonyms and textual definitions are generated automatically, using the grammar described above. The new term is placed automatically into the GO subsumption hierarchy using the ELK reasoner [29] without the need for curator review.

We identified various underlying reasons for most of the discrepancies between the inherent GO chemical ontology and ChEBI. Many of the challenges in making separate ontologies such as the GO and ChEBI parallel result from different fields of study having different viewpoints about the importance of certain characteristics that are represented in their terms and about the axis of classification that they use.

Another issue we encountered in the alignment of the GO and ChEBI is classification by biological role. ChEBI contains a ‘**biological role**’ hierarchy that is separate from its structural, ‘**chemical entity**’ hierarchy. This hierarchy includes terms such as ‘**hormone**’ and ‘**toxin**’, and chemical entities are linked to these roles via the *has*_*role* relationship. Many of these biological roles are also referenced by the GO in terms such as ‘**hormone secretion**’. However, unlike the structural classification axis, using the ‘**biological role**’ hierarchy from ChEBI for the classification of terms within the GO subsumption hierarchy is not straightforward because roles are context-specific. For example, in ChEBI, ‘**acetylcholine**’ *has*_*role* ‘**neurotransmitter**’ and *has*_*role* ‘**hormone**’. This is because in the brain acetylcholine can act as a neurotransmitter, while in other tissues it can act as a hormone. If this role relationship were to be propagated in the GO, that is, asserting ‘acetylcholine secretion’ *is*_*a* ‘neurotransmitter secretion’, the GO would be in error for instances where acetylcholine was secreted but was acting as a hormone. The alignment of the classification of the GO with the ChEBI roles will be undertaken in a separate project.

The GO and ChEBI also differ when chemicals in the GO are classified based on a process in which they are involved. For example, in the GO there are terms like ‘**aspartate family amino acid biosynthetic process**’ that represent the metabolism of amino acid families. These families are not based on the chemical similarities of the amino acids in them, but instead are grouped because they share similar biosynthetic pathways. Participation in related pathways is not essentially a structural feature of the molecules involved, and these processes cannot be represented by the chemical structural hierarchy of ChEBI. However, neither do such groupings easily correspond to ChEBI role terms, since the chemicals are not necessarily active in the pathways involved, as they might, for example, be created by the relevant pathway, and be otherwise quite inert themselves with respect to the operation of the pathway. These pathway-derived chemical classifications will remain in the GO, and for the time being will not be cross-referenced with ChEBI, although such cross-referencing could constitute a task for the future.

We have described here a generic approach to integrating two ontologies that will be used for future projects coordinating the GO with other external ontologies. Before examining relationship concordance, ontology terms should be compared to ensure that the entities that are common to the two ontologies represent the same things and that all of the entities that are implicitly represented in the ontology whose terms are being formally defined are explicitly represented in the external ontology whose terms are being used in the definitions. Next, systematic differences in the construction rationale of the ontologies should be identified and a rational strategy should be put into place where those differences will be retained. Finally, coordinated curation should be used to identify or question relationship differences in the two ontologies. The final process is continuous and mechanisms should be put into place that will allow inconsistencies that crop up to be resolved.

This work is the first part in the integration of ChEBI and the GO. The next stage will be to describe the enzymatic reactions in the GO in terms of the ChEBI entities that participate in them. For example, the molecular function ‘**aspartate dehydrogenase activity**’ is defined as ‘Catalysis of the reaction: L-aspartate + H_2_O + NAD(P)^+^ = oxaloacetate + NH_3_ + NAD(P)H + H^+^’. We intend to leverage the data in Rhea, a manually curated reaction database in which all reaction participants are ChEBI entities [34], to create the logical definitions of GO molecular functions. These definitions will allow us to classify enzymatic reactions automatically based on the chemicals that participate in them; to make better links between biological processes and the reactions that are their parts; and to import new, manually curated reactions directly from Rhea into the GO, and allow them to be automatically classified.

## Methods

### Automatic generation of description logic definitions

To identify the chemical entities referenced by GO terms, we automatically scanned the labels and synonyms for GO terms using the Obol tool [35], looking specifically for references to chemical terms either named in ChEBI or contained within ChEBI’s extensive synonym list. Chemical terms referenced in the GO that were not yet present in ChEBI were identified by manual review of the relevant branches of the ontology (Table 2).

**Table 2:** Initial rules for creation of logical definitions of GO terms

**Term Format**	**Term Genus**	**Relationship to Chemical (X)**
X metabolic process	metabolic process	has_participant some X
X biosynthetic process	biosynthetic process	has_output some X
X catabolic process	catabolic process	has_input some X
X transport	transport	transports_or_maintains_localization_of some X
response to X	response to stimulus	has_input some X
X binding	binding	has_input some X

For each match, we generated a description logic equivalence axiom of the form:

<GO class> EquivalentTo: <Core GO class> <Relationship> some <Chemical>

For example, the GO class with label ‘xanthine biosynthetic process’ was parsed to generate an equivalent class expression ‘biosynthetic process’ and has_output some ‘xanthine’.s

If no chemical entity with the name or synonym ‘X’ could be found in ChEBI, we generated a new class and requested the respective ChEBI entry using the ChEBI web application for term requests (https://www.ebi.ac.uk/chebi/submissions/login). ChEBI curators reviewed requests and either accepted the new entry, added a synonym to an existing ChEBI record or initiated discussion with a GO curator for clarification of the request. These equivalence axioms were maintained as a separate OBO-format “bridge” ontology.

### Making the inherent GO chemical ontology consistent

Initial analysis revealed large disparities not only between the implicit chemical entity hierarchy in the GO and ChEBI, but also within the GO. Therefore we first attempted to achieve internal consistency within the GO, and then proceeded to the larger task of consistency with ChEBI. To do this, we generated a new ontology called ‘GOCHE (GO CHEmicals)’ that used a set of 945 chemical entity classes identified by Obol arranged in a hierarchy determined by relationships in the GO. At this stage we did not yet refer to the ChEBI hierarchy, as the goal was first to reconstruct the implicit chemical classification in the GO and then to compare with ChEBI.

GOCHE was examined and manually edited using the OBO-Edit tool [36]. Chemical term names were made internally consistent within the GO, and ChEBI identifiers were used as the IDs for the chemical terms of the GO chemical ontology. This allowed us to identify the GO chemicals that either needed to be added to ChEBI or resolved with existing ChEBI terms. Chemicals that were missed by Obol but identified during manual review were also added to GOCHE. We then removed all non-structural classifications from the representation of GO chemicals. Next, GO terms were manually inspected and *is*_*a* relationships between chemical-containing terms in the GO were added to the GO chemical ontology. Incorrect relationships were corrected as they were identified.

### Identifying and resolving differences between the GO and ChEBI

**Figure 4:**
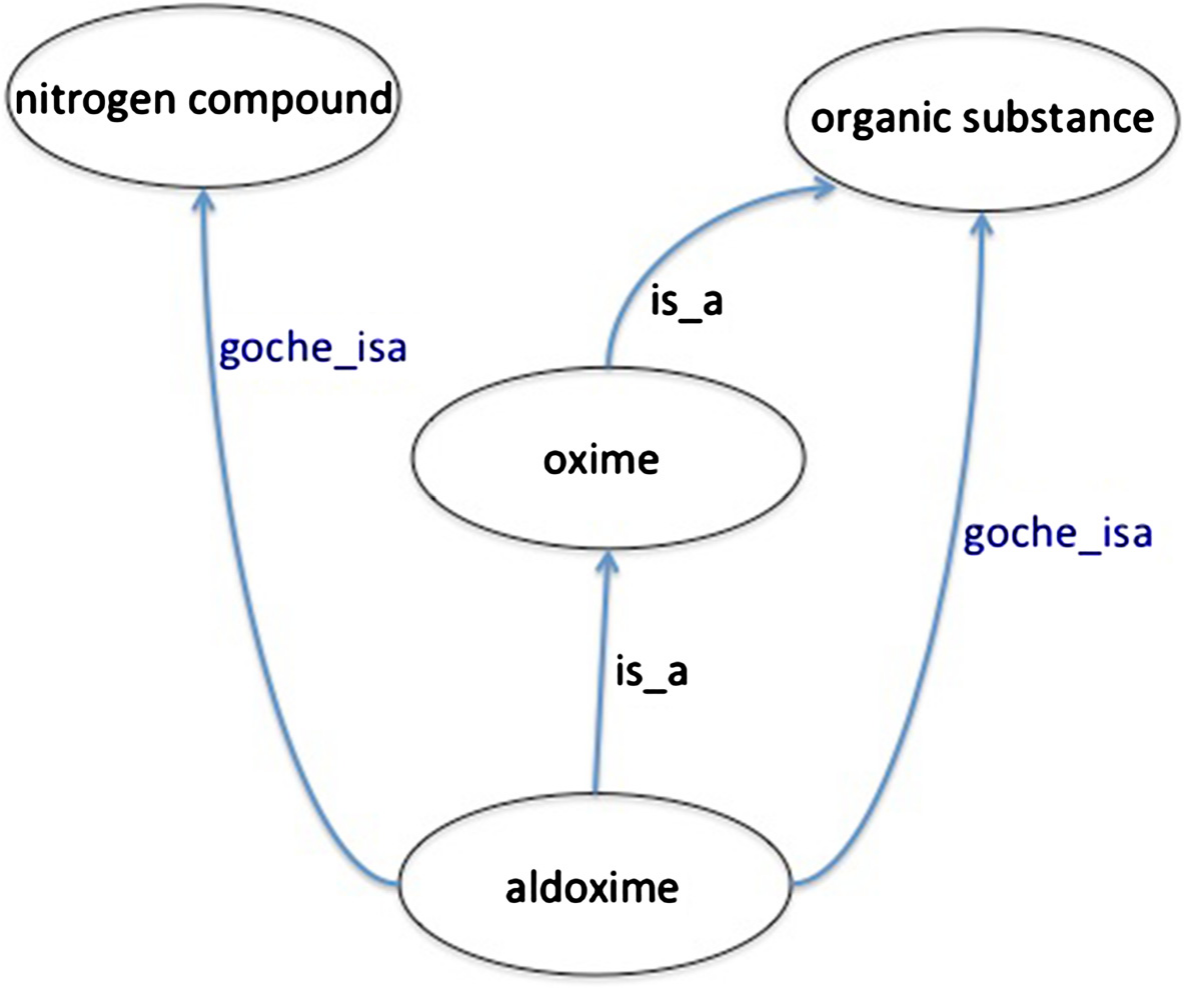
**An example of the combined representation of ChEBI and the GO chemical ontology using OBO-Edit.** These graphical displays were used by ontology curators to identify and resolve representation differences between ChEBI and the GO. In this example aldoxime is a nitrogen compound in the GO, but it is not in ChEBI. Oxime is a term in ChEBI that does not exist in the GO. Only relevant terms and their relationships are shown.

Initially, the GO and ChEBI were compared by simultaneously visualizing the GO chemical ontology and ChEBI in OBO-Edit and distinguishing GO chemical *is*_*a* relationships from ChEBI relationships (Figure 4). Manual inspection was used to identify GO-ChEBI relationship inconsistencies. Errors where the GO had incorrect or missing relationships were fixed in the GO chemical ontology and suspected errors in ChEBI were reported to ChEBI. Next, we used the OBO-Edit reasoner to automatically construct a subsumption hierarchy for the sub-ontologies in the GO that reference chemical entities. This was compared with the existing manually asserted hierarchy. We asserted new links in the GO where suggested by the reasoner and where there was agreement amongst curators. In cases where the curators disagreed with the reasoner, we first manually examined the computable definition for problems. If this definition proved to be correct, then the root cause was a disagreement between ChEBI and the implicit chemical ontology in the GO. In these cases the results were fed back to a wider group for discussion. We also checked for cases where an asserted *is*_*a* link in the GO hierarchy could not be recapitulated by reasoning. These cases arose when a GO editor tried to represent information beyond what could be reasonably inferred directly from ChEBI, and were manually examined and resolved by either deletion or the suggestion of an added relationship to ChEBI.

### Creation of BioChEBI

To handle certain modeling differences between the GO and ChEBI, we created and maintain BioChEBI (http://purl.obolibrary.org/obo/go/extensions/bio-chebi.owl). This ontology imports ChEBI and contains special rules that allow acids and their conjugate bases to be treated as equivalent within the context of in-vivo biological processes. These rules are encoded as OWL general concept inclusion axioms (GCIs) [37], special constructs that specify relationships between broad classes of structures. For example, the GO does not distinguish between citric acid and its three deprotonated forms: citrate(1-), citrate(2-), citrate(3-). In contrast, ChEBI has separate entries for each and links these using ‘is conjugate base of’ and ‘is conjugate acid of’ relations. BioChEBI provides GCIs stating that any biological process that takes ‘citrate acid’ as an input is equivalent to one that takes ‘citrate(1-)’, or one that takes ‘citrate(2-), and so on.

The GCIs for logical definitions containing the ‘has input’, ‘has output’ and ‘transport’ in BioChEBI are generated by the following pseudocode:

FOR each Class X and Y

WHERE

X is SubClassOf ‘is conjugate acid of’ some Y

OR

X is SubClassOf ‘is conjugate base of’ some Y

BEGIN

ADD ‘has participant’ some X = has participant’ some Y

ADD ‘transports’ some X = transports’ some Y

ADD ‘has input’ some X = has input’ some Y

ADD ‘has output’ some X = has output’ some Y

END

These GCIs in BioChEBI allow the direct usage of either acids or their conjugate bases in GO logical definitions, while keeping the original GO semantics. There is no need for new grouping classes and identifiers and the overhead they introduce. Also, we avoid the possible logic inconsistencies by declaring the conjugate base/acid ChEBI terms as equivalent.

As part of the release process for GO, a subset of BioChEBI is generated consisting of only the ChEBI terms used by GO together with descendant classes (http://purl.obolibrary.org/obo/go/extensions/chebi_import.owl). This sub-ontology is itself imported by the extended version of GO (http://purl.obolibrary.org/obo/go/extensions/go-plus.owl) which includes the logical definitions.

### Integration of reasoning into the GO

We use the ELK reasoner to automatically place GO chemical process classes based on their logical definition and placement in ChEBI. This can happen either at the time of class creation, or alternatively after a new release of ChEBI in which the placement of chemical entity classes moves. In either case, the procedure is as follows: the reasoner is invoked via the OWL API (Application Programmer Interface) and the most specific superclasses are obtained. The resulting relationship is asserted into the main GO editors ontology as a SubClassOf axiom, and this axiom is tagged with an axiom annotation marking it as having been inferred by an automated procedure. If a SubClassOf axiom has been tagged in this way from a previous inference, and it is no longer a valid inference, then it is removed. This allows the GO chemical process hierarchy to stay in sync with ChEBI. In both cases, a report is created showing new automated links added, and automated links previously added that have been removed.

## Competing interests

The authors declare no competing interests.

## Authors’ contributions

CJM and MB worked on the initial parsing of GO terms into their chemical components and processes which identified misalignments between GO and ChEBI. DPH, NA, MB, CB, TZB, HJD, ME, RF, MAH, JH, PdM, CJM, GO, PR and JL attended meetings and participated in discussions of strategies to align the GO and ChEBI. DPH, TZB, HJD, RF, MAH, PR, CJM, HD and JL worked on changes to the Gene Ontology. NA, ME, NK, PdM, GO, CS and ST worked on changes to ChEBI. HD and CJM wrote the software and generated the files used in the project. DPH drafted the manuscript. All authors contributed to editing the manuscript. All authors approved of the final version of the manuscript.
